# Rehabilitation and return to activity after platelet‐rich plasma injections in chronic tendinopathies: Consensus from international experts

**DOI:** 10.1002/pmrj.70087

**Published:** 2026-01-17

**Authors:** Vincent Gremeaux, Martin Lamontagne, Valérie Bélanger, Etienne Dalmais, Eric Noël, Alain Frey, Paul Ornetti, Fadoua Allali, Jimmy Gross, F. Michel, Hervé Bard, Jean‐François Kaux, the GRIIP (Groupe de Recherche International sur les Injections de Plaquettes; International Research Group on Platelet Injections)

**Affiliations:** ^1^ Sport Medicine Unit, Division of Physical Medicine and Rehabilitation, Swiss Olympic Medical Center Lausanne University Hospital Lausanne Switzerland; ^2^ Department of Physiatry Hospital Center, Montreal University Montreal Québec Canada; ^3^ Department of Medicine, Physiatry service program Quebec City University Hospital Center Quebec City Québec Canada; ^4^ Centre d'Orthopédie et de Traumatologie du Sport Bassens France; ^5^ Santy Orthopedic Center Lyon France; ^6^ Sport Medicine Department Centre Hospitalier Intercommunal Poissy/St Germain Poissy France; ^7^ Service de Rhumatologie, Plateforme d'Investigation Technologique CIC 1432 Module Plurithématique Dijon France; ^8^ Inserm CAPS 1093 Université de Bourgogne Dijon France; ^9^ Department of Rheumatology El Ayachi Hospital Salé Morocco; ^10^ Institut de Rhumatologie Interventionnelle (IRI) Paris France; ^11^ Centre Locomoteur de Vincennes (CLV) Vincennes France; ^12^ Department of Physical Medicine and Rehabilitation Jean‐Minjoz University Hospital Besançon France; ^13^ Rhumatologue, Cabinet Vaudoyer Paris France; ^14^ Physical and Rehabilitation Medicine, SportS^2^, FIFA Medical Centre of Excellence, FIMS Clinical Centre of Sports Medicine, ReFORM IOC Research for Prevention of Injury and Protection of Athlete Health University of Liège and University Hospital of Liège Liège Belgium

## Abstract

**Background:**

The effectiveness of platelet‐rich plasma (PRP) injections in chronic tendinopathies remains debated. Although the product's characteristics play a role, the impact of postinjection recommendations remains poorly investigated.

**Objective:**

To establish principles and guidelines for post‐PRP injection rehabilitation and return to activity/sports.

**Design:**

We conducted a Delphi survey based on a “recommendations by formal consensus” methodology. Three clinicians and researchers of the GRIIP (International Research Group on Platelet Injections), experts in sports medicine and rehabilitation, performed a comprehensive literature review of MEDLINE, searching for (1) rehabilitation protocols after PRP injection for chronic tendinopathy and (2) fundamental studies of tendon healing. This review highlighted the main points to be clarified to establish a standardized post‐PRP injection protocol. Fourteen points were identified, grouped into three dimensions (immediate postprocedure, rehabilitation, and follow‐up and resumption of sports/activity). With a modified Delphi method, the propositions were submitted to a panel of 23 experts from five French‐speaking countries. The recommendations were classified as appropriate or not appropriate, with strong or relative agreement, or uncertain when consensus was not reached.

**Results:**

Ten recommendations (postprocedure avoidance of nonsteroidal anti‐inflammatory drugs, rehabilitation principles and timeline, follow‐up) were classified as appropriate: six with strong and four with relative agreement; four propositions regarding immediate postprocedure were deemed uncertain.

**Conclusion:**

Agreement was reached for 10 of the 14 recommendations but with low level of evidence, mainly based on experts' opinion. This work should help standardize a post‐PRP injection protocol for chronic tendinopathies, which will minimize bias due to variations in rehabilitation protocols in further studies.

## INTRODUCTION

Platelet‐rich plasma (PRP) injections aim to stimulate an anabolic response in a tendon whose intrinsic healing ability appears to be deficient.^1^ The various molecules contained in PRP aim to induce a biological response in the tissue to promote healing.^2^ In addition, the tendon must be mechanically stimulated with a progression of exercises to achieve a synergistic effect. This mechanical load applied to the tendon tissue is transformed into electrochemical activity, inducing a biological response via cellular “mechanotransduction.”^3^, ^4^ The appropriate mechanical stimuli produced by exercise induces cellular and tissue remodeling conducive to healing.^5^, ^6^, ^7^

There are an increasing number of publications evaluating the efficacy of PRP injections for tendinopathy, with contrasting results.^8^, ^9^, ^10^, ^11^ One of the main issues usually discussed concerns the lack of standardization of the PRP content.^12^, ^13^ Postinjection immediate care and rehabilitation programs are also rarely described, even though they have a major influence on clinical and functional results after orthobiologic interventions.^3^, ^4^ A few nonrandomized studies have had interesting results when combining PRP treatment with a specific rehabilitation protocol.^14^, ^15^, ^16^, ^17^ To our knowledge, no study has assessed whether a specific rehabilitation program following this type of treatment leads to superior results, nor compared different postinjection protocols. In such circumstances, when scientifically sound facts are lacking, the Delphi method relying mainly on expert opinion is often chosen to obtain consensus. This methodology has been recently used for the development of recommendations on orthobiologic treatments for osteoarthritis.^18^, ^19^

This project aimed to develop consensus on a protocol regarding post‐PRP injection rehabilitation and return to activity/sports.

## METHODS

The methodology was based on recommendations from the French health authority,^20^ inspired by the Delphi method. A pilot group conducted a comprehensive literature review of all published therapeutic trials, randomized or nonrandomized studies, and systematic reviews/meta‐analyses focusing on the rehabilitation protocol after PRP injection for chronic tendinopathy, as well as fundamental studies concerning tendon healing. The pilot group consisted of three French‐speaking medical physicians (V.G., M.L., J.F.K.) specialized in physical medicine and rehabilitation (PMR) and sports medicine who work in public or private medical centers and are experienced in research and PRP use. The physicians were from three countries (Belgium, Canada, and Switzerland) and are members of GRIIP (International Research Group on Platelet Injections). Literature was searched in MEDLINE and included articles indexed before October 2022. The main elements of this review were presented at the first GRIIP International conference in Paris in January 2023.^21^ Then, the group set video meetings to pinpoint the main items to be clarified in a standardized post‐PRP injection rehabilitation protocol. Fourteen points were identified, grouped into three dimensions, namely immediate postprocedure, rehabilitation, and follow‐up and return to sports/professional activities. These propositions were approved by an independent reading group (E.D., V.B.).

A second group of 23 experts was selected from GRIIP members and their contacts and included 1 orthopedic surgeon, 4 rheumatologists, 8 general practitioners, and 10 PMR physicians, the last two groups practicing mainly as sports medicine physicians, from five French‐speaking countries (Belgium, Canada, France, Morocco, and Switzerland).

All experts used a 9‐point Likert scale to vote anonymously for each of the 14 recommendations through an online survey, and they could leave specific comments. The first voting round took place from March 1 to April 30, 2024. The pilot group then analyzed the results and comments, and three recommendations were slightly rephrased. The modified survey was resent to the 23 experts, with a short summary indicating the first round's median score and range for each recommendation. This second round ran from June 15 to August 15, 2024. The pilot group performed a debriefing meeting after this second round to identify issues to be discussed, mainly regarding the propositions for which no consensus was reached. Finally, the 23 experts were asked in a third round, running from September 15 to October 15, 2024, to rank the recommendations for each dimension in order of importance.

### Rating of recommendations

Recommendations were scored on a Likert scale from 1 to 9, with 1 meaning “totally inappropriate” or “total disagreement” and 9 meaning “totally appropriate” or “total agreement”; the value of 5 indicated “indecision” (Table 1). Scores were pooled, and medians were calculated. Evaluation accordance was mainly based on the median^20^: a proposition was considered appropriate with a median of ≥7, with strong agreement for scores from 7 to 9 and relative agreement for scores from 5 to 9. In contrast, a recommendation was considered inappropriate with a median of ≤3, with strong agreement for scores from 1 to 3 and relative agreement for scores from 1 to 5. Propositions were considered uncertain, reflecting indecision, with a median of 4–6 and scores from 1 to 9 and uncertain without consensus in all other situations (Table 1).

**TABLE 1:** Rating of recommendations based on median and distribution of the scores (from^20^, ^22^).

Proposition qualification	Level of agreement	Median	Min–Max
Appropriate	Strong	≥ 7	7–9
	Relative	≥ 7	5–9
Inappropriate	Strong	≤ 3	1–3
	Relative	≤ 3.5	1–5
Uncertain	Indecision	4 < MED <6.5	1–9
	No consensus	All other situations	

In addition, a level of evidence was assigned to each recommendation, depending whether it was based on studies and their design or on expert opinion (from 1A, best evidence to 5, weak evidence).^22^

## RESULTS

After the first round, consensus was reached on recommendation number 10, which was therefore considered appropriate with strong consensus and was not rated again during the second round. The evolution of the expert rating is shown in Tables 2, 3, 4, 5 depict the median, distribution of scores, appropriateness, inappropriateness, uncertainty, and level of agreement for each recommendation.

**TABLE 2:** Evolution of the experts' advice through the 2 rounds for each question.

	First round	Second round
Appropriate with strong agreement	10	5, 6, 10, 11, 13, 14
Appropriate with relative agreement	9, 11, 13	7, 8, 9, 12
Uncertain	1, 3, 4	1, 2, 3, 4
Uncertain without consensus	2, 5, 6, 7, 8, 12, 14	

**TABLE 3:** Appropriateness, level of agreement, and level of evidence for propositions 1–5 (immediate postprocedure).

Question	Immediate postprocedure	Median	Min	Max	Proposition	Level of agreement
1	The application of ice in the immediate aftermath of an intra‐tendinous injection of PRP may alter platelets activation	6	2	7	Uncertain	Indecision
2	Partial unloading is recommended after PRP injection for chronic tendinopathy of the lower limb	8	6	9	Uncertain	Indecision
3	Wearing an orthosis or an immobilization boot is recommended after a PRP injection for chronic tendinopathy of the lower limb	5	1	8	Uncertain	Indecision
4	Wearing an immobilization brace is recommended after an intra‐tendinous injection of PRP for chronic tendinopathy of the upper limb	5	1	8	Uncertain	Indecision
5	NSAIDs should be avoided for the first few weeks after a PRP injection for chronic tendinopathy	9	8	9	Appropriate	Strong

Abbreviations: NSAID, nonsteroidal anti‐inflammatory drug; PRP, platelet‐rich plasma.

**TABLE 4:** Appropriateness, level of agreement, and level of evidence for propositions 6–10 (rehabilitation phase).

Question	Rehabilitation phase	Median	Min	Max	Proposition	Level of agreement
6	Tendon rehabilitation should be started quickly (after 5–10 days) after an intratendinous injection of PRP for chronic tendinopathy	8	7	9	Appropriate	Strong
7	Rehabilitation after intratendinous injection of PRP should include stretching (except for certain compression tendinopathies)	8	5	9	Appropriate	Relative
8	Rehabilitation after intratendinous injection of PRP should include sub‐painful isometric work (VAS ≤3/10 during and for 24 h after the session)	9	5	9	Appropriate	Relative
9	Rehabilitation after intratendinous injection of PRP should include subpainful dynamic work (concentric and/or eccentric; VAS ≤3/10 during and for 24 h after the session).	9	5	9	Appropriate	Relative
10	The final phase of rehabilitation should include specific exercises related to the patient's job or sport	9	7	9	Appropriate	Strong

Abbreviations: PRP, platelet‐rich plasma; VAS, visual analog scale.

**TABLE 5:** Appropriateness, level of agreement, and level of evidence for propositions 11–14 (follow‐up and return to sports/professional activities).

Question	Follow‐up and return to sports/professional activities	Median	Min	Max	Proposition	Level of agreement
11	A gradual return to professional and/or sporting activities is considered when the clinical examination reveals little or no pain (VAS <3/10).	9	7	9	Appropriate	Strong
12	Monitoring should include an algo‐functional score (eg, VISA‐A, VISA‐P)	9	6	9	Appropriate	Relative
13	Imaging is not needed to decide whether to return to work and/or sport after tendon rehabilitation can be achieved	9	7	9	Appropriate	Strong
14	A second injection may be suggested in case of partial improvement after 3 months	9	7	9	Appropriate	Strong

Abbreviations: VAS, visual analog scale; VISA‐A, Victorian Institute of Sport Assessment‐Achilles; VISA‐P, Victorian Institute of Sport Assessment‐Patellar Tendon.

After the third round, the priority of recommendations was as follows: for immediate postprocedure, R5 was ranked first by all the experts, and R2 was most often cited as the second priority, followed by R1, R3, and R4. Regarding rehabilitation phase, R6 was the most cited as 1st rank, followed by R8 and R10, R9, and R7. Finally, for follow‐up, R11 was the favored recommendation, followed by R13, R14, and R12.

## DISCUSSION

PRP injection in chronic tendinopathy gained popularity with the growing interest in regenerative medicine. However, it remains one piece in the puzzle of tendinopathy treatment, whose cornerstone remains progressive loading to induce positive adaptation, with a timeframe based on the model of biological healing of soft tissues. Thus, having standardized principles and guidance on postprocedure care is paramount to optimize efficiency, in addition to clarifying the best factors for the PRP composition itself (concentration, leucocytes or not, volume).^23^

The objective of this work was to set the principles and guide clinicians in post‐PRP injection rehabilitation and return to activity/sport based on literature evidence and consensus among experts. Several recommendations emerged from this work, despite certain limitations and methodological pitfalls inherent to consensus studies. In addition, cost–benefit studies would be of interest.

Ten recommendations were classified as appropriate, six with strong agreement and four relative agreement (postprocedure avoidance of nonsteroidal anti‐inflammatory drugs [NSAIDs], rehabilitation principles and timeline, follow‐up). Four propositions regarding the immediate postprocedure were deemed uncertain. The recommendations failing to reach consensus concerned the immediate postprocedure for various reasons discussed next, and the impact of those factors on the final clinical results remain to be studied. Conversely, there was consensus regarding the rehabilitation framework and criteria to return to sports or physically demanding professions. However, this consensus remains based on expert opinion because of only few data regarding the optimal time to resume different kinds of exercises as well as professional/sports activities, as discussed subsequently.

### First dimension: immediate postprocedure (recommendations 1–5)

R1: The application of ice in the immediate aftermath of an intratendinous injection of PRP may alter platelet activation: *uncertain with indecision, level of evidence 3A*.

There are still debates regarding the potential deleterious effect of cold in soft‐tissue injuries and platelet activation,^4^ which may explain the uncertainty and variable priority of this recommendation (ranked third). A recent systematic review did not find any study of humans regarding effects of cryotherapy on tendon healing,^24^ and only one study of mouse patellar and calcaneal tendon injury by needle penetration showed reduced renal prostaglandin E2 level by half within 3.5 hours after a 30‐minute postinjury cryotherapy session with refrigerant gel.^25^ In addition, the early inflammation phase after a soft‐tissue injury has been found beneficial by attracting blood cells such as platelets, neutrophils, and monocytes to the site of injury by proinflammatory cytokines. However, whether ice application leads to a temperature decrease to a sufficient extent to influence these processes and thus potentially modify PRP‐induced healing abilities remains unclear. Current studies mainly investigate the effects of cryotherapy on muscle recovery after sports‐induced muscle damage.^3^ However, Sussman et al. found that in only 20% of analyzed studies, cryotherapy was recommended as part of the rehabilitation after regenerative and orthobiologic treatments,^4^ and only one study of patients with diabetes reported changes in plasma platelet activation after 60‐second cold stress induced by arm immersion in ice cold water.^26^

R2, R3, and R4: Partial unloading is recommended after PRP injection for chronic tendinopathy of the lower limb (R2). Wearing an orthosis or an immobilization boot (R3) or brace (R4) is recommended after PRP injection for chronic tendinopathy of the lower limb (R3) or upper limb (R4): *uncertain with indecision, level of evidence 3A*.

The potential risk of early weight‐bearing for tendon after a needle procedure is poorly documented. Housner et al. reported a tendon rupture in a study investigating the effects of ultrasound‐guided needle fenestration in recalcitrant patellar tendinopathy.^27^ In their systematic review, Sussman et al. found that 14 of 60 studies recommended not moving the tendon for up to 60 minutes after the procedure, some recommending using slings or weight‐bearing restrictions for the lower limb.^4^ Most authors recommend a period of absolute or relative rest during the inflammatory phase, although the recommendations still vary.^14^, ^16^, ^17^ Although the benefits and length of a short immobilization period remain unknown, some studies showed accelerated beneficial changes in mouse tendons with early mobilization after Achilles rupture.^28^ In humans, only one review was conducted after minimally invasive repair of Achilles tendon rupture, and immediate weight‐bearing in a functional brace, together with early mobilization, appeared to be safe with superior outcomes.^29^ Among our panel, for those favoring immobilization and delayed weight‐bearing for lower limbs, the rationale was based mainly on pain management or controlling a potential inflammation flare‐up, rather than fear of a serious adverse effect.

R5: NSAIDs should be avoided for the first few weeks after PRP injection for chronic tendinopathy: *appropriate with strong consensus, level of evidence 1B*.

NSAIDs are known to inhibit the platelet degranulation process and thus may alter the PRP effects by inhibiting cyclooxygenase‐1 and/or 2, key enzymes in inflammation regulation. In 2004, Kariyazono et al. examined the effects of aspirin, cilostazol, and ramatroban on PRP aggregation induced by adenosine diphosphate, collagen, and arachidonic acid and found that these drugs inhibited PRP aggregation dose‐dependently.^30^ More recently, Leach et al. reviewed the dose and time effect of various NSAIDs on platelet aggregation inhibition, showing that cyclooxygenase‐1 inhibitors significantly altered platelet aggregation for 6 to 48 hours, with negligible effects for cyclooxygenase‐2 inhibitors and acetaminophen.^31^

All these fundamental data probably explain why this recommendation was ranked as first priority by our experts. No studies have investigated the potential in vivo role of NSAIDs in altering the effects of PRP in tendon procedures, but in vitro studies seem to recommend avoiding these drugs at least during the inflammation phase of the soft‐tissue healing process.^23^ From the experts of this work, a 2‐week period seems to be the most consensual recommendation.

### Second dimension: Rehabilitation phase (recommendations 6–10)

R6: Tendon rehabilitation should be started quickly (after 5–10 days) after an intratendinous injection of PRP for chronic tendinopathy: *appropriate with strong consensus, level of evidence 4*.

R7: Rehabilitation after intratendinous injection of PRP should include stretching (except for certain compression tendinopathies) (R7): *appropriate with relative consensus, level of evidence 4*; subpainful isometric work (visual analog scale score ≤3/10 during and for 24 hours after the session) (R8): *appropriate with relative consensus, level of evidence 4*; subpainful dynamic work (concentric and/or eccentric; visual analog scale [VAS] score ≤3/10 during and for 24 hours after the session) (R9): *appropriate with relative consensus, level of evidence 4*.

R10: The final phase of rehabilitation should include specific exercises related to the patient's job or sport: *appropriate with strong consensus, level of evidence 4*.

There is consensus on the importance of implementing specific exercises increasing the mechanical stress on the treated tendon.^4^ In line with this consensus, experts ranked R6 as number one. The progression model remains an estimate and may vary according to numerous factors, including the patient's age, co‐morbidities, sports experience, occupation, and the severity of the injury.^3^, ^7^ In 2009, Kon et al. found that among 20 patients treated for chronic patellar tendinopathy with three PRP injections, those who did not follow the postprocedure stretching and strengthening program showed poorer outcomes.^32^ Early controlled‐load applications after an orthobiologic procedure were then found to accelerate the repair process, reduce pain, and increase tissue regeneration.^6^, ^33^

There is currently no consensus on the optimal type and timing of stretching exercises in tendinopathy, whether PRP injection is performed or not.^34^ Whether the transient reduction in tendon perfusion found in passive stretching^35^ may influence the outcome after an orthobiologic injection if too intense or if started too early is unknown, which may explain why experts ranked R7 with a lower level of priority.

In the Sussman et al. review,^4^ strengthening exercises began 2 weeks after the procedure; however, >50% of the studies did not specify the type of muscle contraction (isometric, concentric, or eccentric), and none specified whether open or closed kinetic chain exercises must be prioritized. However, a graduated strengthening program, from isometric to concentric and then eccentric exercises is usually suggested, in line with the main framework of state‐of‐the‐art rehabilitation protocols for chronic tendinopathy.^36^ Therefore, the pilot group suggested this progression and the experts recommended it. However, there are still controversies on the progression steps. In most studies describing their protocol after PRP injections, eccentric exercise was introduced after 5–6 weeks,^14^, ^15^, ^17^ whereas Kaux et al. suggested early submaximal eccentric exercises in patellar tendinopathy 1 week after injection, based on animal models.^37^ Eccentric contractions are often feared because they are known to produce a greater amount of tension than concentric muscle contractions, and they may lead to muscle damage and delayed pain, commonly defined as delayed‐onset muscular soreness. However, although muscle or tendon tear often results from a sudden uncontrolled heavy eccentric contraction, submaximal controlled and progressively increasing eccentric exercise seems safe for rehabilitation.^38^ Finally, in tendinopathy, the contraction mode as measured by increased tendon stiffness has been found superior to the others.^39^ In addition, the threshold for an acceptable level of pain on a VAS during exercises also varies but is generally consistent with the “pain‐guided reactivation” model suggested in chronic tendinopathy.^36^, ^40^

Finally, there was high agreement among experts on the importance of a professional or sports‐specific phase when tolerated load is sufficient, in line with best practice recommendations in tendon rehabilitation and rehabilitation in other fields of sports medicine widely studied, such as return to sports after anterior cruciate ligament surgery.^36^, ^41^

### Third dimension: follow‐up and return to sports/professional activities (recommendations 11–14)

R11: A gradual return to professional and/or sporting activities is considered when the clinical examination reveals little or no pain (VAS score <3/10): *appropriate with strong consensus, level of evidence 4*.

R12: Monitoring should include an algo‐functional score: *appropriate with relative consensus, level of evidence 5*.

R13: Imaging is not needed to decide whether a return to work and/or sport after tendon rehabilitation can be achieved: *appropriate with strong consensus, level of evidence 3B*.

R14: A second injection may be suggested in case of partial improvement after 3 months: *appropriate with strong consensus, level of evidence 4*.

R11 and R13 were deemed the most important return to sport/activity recommendations, supporting the acknowledged importance of clinical examination over imaging findings.^42^, ^43^

In the tendon rehabilitation literature, the use of algo‐functional scores is common. However, among experts, some clinicians argued that the recommendation “possibly including an algo‐functional score” may be more appropriate, given the time required to complete the questionnaire, sometimes difficult to fit in during clinical routine practice. However, experts all acknowledged that the score would be the optimal follow‐up tool.

Regarding criteria to return to sports or physically demanding professional activity, there was consensus to base the decision on clinical examination, in line with literature on many chronic conditions or healing after an acute condition/surgery, for which a mismatch between imaging and symptoms is common.^42^, ^43^

Finally, the recommendation to wait 3 months before considering a new injection when only partial improvement is obtained seems consistent with fundamental data suggesting a minimal rehabilitation period of 3 months.^3^, ^36^ In addition, Kaux et al. demonstrated no further improvement when a second injection was performed 2 weeks later in patellar chronic tendinopathy.^44^ From the experts opinion, the most widely accepted definition of partial improvement appears to be an improvement in pain or function of between 50% and 70%.

## LIMITS

One limitation of this work is the methodology itself, being an expert opinion‐based consensus, which primarily aims to bridge the gaps in literature. In addition, all experts were from French‐speaking countries, which may suggest cultural‐based bias and limit the scope of the recommendations at an international level. However, although experts were mainly from European countries (Belgium, France, Switzerland), four were from Canada and two from Morocco, thus representing different health care systems and medical cultures. Moreover, the sample included 1 orthopedic surgeon, 4 rheumatologists, and 18 sports medicine physicians who were originally general practitioners or PMR doctors. Finally, almost 2 years elapsed between the initial literature review and the end of the two rounds as well as the analysis of the results. However, we performed an additional literature search after the third round, which identified similar studies in the field of osteoarthritis^45^, ^46^ but none in the field of tendinopathy.

## CONCLUSION

This work resulted in expert agreement on 10 of 14 recommendations regarding PRP injection for chronic tendinopathies. These essential principles should be integrated in protocols for after PRP injection in chronic tendinopathies. There remains some uncertainty about the use of ice and initial loading right after the procedure owing to lack of data in the literature and different cultural beliefs among practitioners. Immediate post‐PRP interventions may affect clinical results but likely less significantly than interventions during the rehabilitation phase itself. These recommendations should help in designing more consistent postinjection protocols and by doing so, better isolate the effects of PRP composition on patient outcomes in future clinical research studies.

### Findings

The results of studies on the use of PRP injection in tendinopathies remain conflicting. The reasons usually discussed are the lack of characterization of the product or the pathology. Postinjection management is rarely described, although it may influence results.

### Implications

This work resulted in the description of the main outline of a standardized PRP post‐injection procedure with expert consensus, which will allow for limiting the biases associated with postprocedure care in future studies.

### Caution

In the absence of literature regarding this topic, these recommendations are mainly based on expert opinion and may vary by patient characteristics and sociocultural factors.

## DISCLOSURE

The authors have no conflict of interest to declare.
